# Chagas disease: a report of 17 suspected cases in Japan, 2012–2017

**DOI:** 10.1186/s41182-019-0168-3

**Published:** 2019-06-13

**Authors:** Kazuo Imai, Kazuhisa Misawa, Morichika Osa, Norihito Tarumoto, Jun Sakai, Kei Mikita, Yusuke Sayama, Yuji Fujikura, Akihiko Kawana, Takashi Murakami, Shigefumi Maesaki, Sachio Miura, Takuya Maeda

**Affiliations:** 1 Department of Infectious Disease and Infection Control, Saitama Medical University, Saitama, Japan; 2 Division of Infectious Diseases and Pulmonary Medicine, Department of Internal Medicine, National Defense Medical College, Saitama, Japan; 3 Department of Infectious Diseases, Keio University School of Medicine, Tokyo, Japan; 4 Central Blood Institute, Blood Service Headquarters, Japanese Red Cross Society, Tokyo, Japan; 5 Department of Microbiology, Saitama Medical University, 38 Morohongo, Moroyama-machi, Iruma-Gun, Saitama 350-0495 Japan; 6 Tokyo College of Medical Technology, Tokyo, Japan

**Keywords:** Chagas disease, Immigrants, Epidemiology, Cardiomyopathy, Japan

## Abstract

**Background:**

There are no reports on the prevalence of Chagas disease in Japan. Furthermore, screening programs and access to diagnosis and treatment have not been established. This study aimed to clarify the prevalence of Chagas disease among suspected cases in Japan and provide the reference data required for disease control.

**Methods:**

Seventeen patients with suspected Chagas disease in Japan between 2012 and 2017 were included in the study. Patients were diagnosed with Chagas disease based on the two different serological tests for antibodies to *Trypanosoma cruzi*. Real-time polymerase chain reaction assay and blood culture techniques were performed to confirm *T. cruzi* parasitemia.

**Results:**

Of the 17 patients, 11 (64.7%) were immigrants from Latin America. Ultimately, 6 patients (35.3%) were diagnosed with Chagas disease. Of these 6 patients, median age was 53.5 years, 5 patients were immigrants from Latin American, and 1 was Japanese who had a congenital infection. *T. cruzi* parasitemia was confirmed in 4 patients (66.7%), and 5 (83.3%) were in the chronic phase (Chagas cardiomyopathy, 4; megacolon, 1). Two patients (33.3%) commenced benznidazole treatment.

**Conclusion:**

Our study showed that some patients of Chagas disease living in Japan are already in the chronic phase at diagnosis because of substantial diagnostic delays. Further epidemiological studies on the prevalence of Chagas disease and systematic screening programs for the Latin American population are needed.

## Background

The epidemiology of Chagas disease, caused by the parasite *Trypanosoma cruzi*, has radically changed with the increasing numbers of immigrants from Latin America, where the disease is endemic. Approximately 250,000 immigrants from Latin America are living in Japan, and in 2010, it was estimated that over 3000 immigrants may have latent Chagas disease infection [1]. In endemic areas, the main route of transmission is by an insect vector known as *Triatominae* (kissing bugs). Imported cases can potentially spread the infection via non-vector-borne routes such as blood transfusion, organ transplantation, and mother-to-child transmission. In fact, cases of local transmission in non-endemic countries have been described worldwide [2]. The control of Chagas disease is a challenge that requires continuous public health programs.

An epidemiological study found that the prevalence of Chagas disease was 0.017% among 18,076 blood donors who were at risk of Chagas disease in Japan [3]. Asymptomatic carriers among donors born in endemic areas and cases of congenital Chagas disease have been reported in Japan [4]. The present study aimed to clarify the prevalence of Chagas disease among suspected cases in Japan as a preliminary investigation, and highlights the importance of identifying the clinical characteristics of patients, especially in non-endemic countries.

## Material and methods

Subjects were 17 patients with suspected Chagas disease based on clinical findings (cardiomyopathy, with baseline electrocardiogram or echocardiography abnormalities; gastrointestinal disorders, megacolon, or megaesophagus) and life history (resident in Japan; those with a travel history to Latin America, those born or raised in Latin America, and those whose mother was born or raised in Latin America) from 2012 to 2017. Chagas disease was confirmed with two different serological tests for antibodies to *T. cruzi*. Specifically, the first serological screening was performed using an immunochromatographic test (ICT; Trypanosoma Detect™ Rapid Test, InBios International Inc., Seattle, WA) according to the manufacturer’s protocol. Positive results were subsequently confirmed via an enzyme-linked immunosorbent assay (ELISA; Chagas Detect™ Fast ELISA, InBios). Additionally, real-time polymerase chain reaction assay was used to detect the presence of *T. cruzi* DNA in blood samples [5]. Parasitological diagnosis using the blood culture technique for *T. cruzi* was also performed.

## Results

Clinical characteristics of the patients and the results of diagnostic tests are summarized in Table 1. Median age of the 17 patients was 50 years; among them, 11 patients (64.7%) were immigrants from Latin America (Brazil, 8; Peru, 1; Bolivia, 1) while 6 (35.3%) were Japanese patients suspected of having *T. cruzi* infection based on either travel history (Ecuador, 1; Latin American countries, 3), birthplace (Peru, 1), or potential mother-to-child infection (Japan, 1). Of the 17 patients, 11 (64.7%) had cardiac disorders including arrhythmia, heart failure, myocarditis, and cardiomyopathy, and 1 (5.9%) had megacolon.

**Table 1:** Clinical characteristics and diagnostic results among cases with suspected and confirmed Chagas disease

Case	Year	Sex	Country of origin	Presumed country of infection	Clinical finding	Diagnosis	Serodiagnosis		qPCR (parasites/mL)	Culture	Treatment
							ICT	ELISA			
1	23	F	Japan	Ecuador (travel)	Myocarditis Heart failure	–	–	NA	NA	NA	NA
2	52	F	Japan	Latin America (travel)	Myopathy	–	–	NA	NA	NA	NA
3	50	M	Brazil	Brazil	Cardiomyopathy	–	–	NA	NA	NA	NA
4	40	M	Brazil	Brazil	Arrhythmia	–	–	NA	NA	NA	NA
5	47	M	Brazil	Brazil	LVH	–	–	NA	NA	NA	NA
6	NA	NA	Japan	Latin America (travel)	Malaise	–	–	NA	NA	NA	NA
7	NA	NA	Japan	Latin America (travel)	Periorbital edema	–	–	NA	NA	NA	NA
8	50	M	Brazil	Brazil	Cardiomyopathy Arrhythmia	–	–	NA	NA	NA	NA
9	69	M	Peru	Peru	Myocarditis Heart failure	–	–	NA	NA	NA	NA
10	14	F	Japan	Peru (birth place)	Myocarditis Heart failure	–	–	NA	NA	NA	NA
11	16	F	Brazil	Brazil	Myocarditis Heart failure	–	–	NA	NA	NA	NA
12	13	F	Japan	Japan (congenital)	Megacolon	Chronic (digestive)	+	× 12,800	3.2	+	Bez
13	49	M	Bolivia	Bolivia	None	Indeterminate	+	× 800	0.1	NA	–
14	55	F	Brazil	Brazil	Heart failure Arrhythmia	Chronic (cardiac)	+	× 12,800	–	–	Bez
15	52	F	Bolivia	Bolivia	Arrhythmia	Chronic (cardiac)	+	× 50	1.6	NA	–
16	64	F	Brazil	Brazil	Heart failure Arrhythmia	Chronic (cardiac)	+	× 64,00	–	NA	–
17	68	M	Brazil	Brazil	Heart failure	Chronic (cardiac)	+	× 1600	0.4	+	–
					Arrhythmia						
					Ventricular						
					aneurysm						

*M* male, *F* female, *NA* not available, *ICT* immunochromatographic test, *LVH* left ventricular hypertrophy, *Bez* benznidazole

Samples from 6 patients (35.3%) were positive on both serological tests, and the patients were subsequently diagnosed with Chagas disease. Of these 6 patients, 4 (66.7%) had *T. cruzi* DNA in whole blood samples in the range 0.1–3.2 parasites/mL. Blood cultures of samples from 2 patients (33.3%) were also positive. The median age of the patients with confirmed Chagas disease was 53.5 years; 5 patients were immigrants from Latin America (Brazil, 3; Bolivia, 2), and 1 was Japanese. This patient had a congenital infection and was born in Japan to a Bolivian mother; his mother was diagnosed with Chagas disease at the same time [4]. Among the clinical findings, chronic-phase Chagas disease was diagnosed in 5 patients (83.3%), while the phase could not be determined in 1 asymptomatic patient. Chagas cardiomyopathy was present in 4 patients (66.7%) and megacolon in 1 patient (16.6%). Factors other than Chagas that contributed to the cardiac and gastrointestinal disorders were excluded. Benznidazole, used for the treatment of Chagas disease at our hospitals, was initiated in 2 patients (33.3%).

## Discussion

Our study provides the first insight into the population characteristics of patients with Chagas disease in Japan. Surprisingly, up to 83.3% of patients with Chagas disease developed chronic complications, including a patient infected by mother-to-child transmission. The most recent series of reports on chronic Chagas disease in non-endemic countries show that mean ages are in the range 35–41 years, 78.9–97.0% of cases originate in Bolivia, and 18.6–49.0% of patients have Chagas cardiomyopathy at the initial diagnosis [6–11]. The clinical profiles including the diagnosis of younger patients and lower rates of complications differed from our results. These differences could reflect the substantial delays in the diagnosis of Chagas disease in Japan, which are influenced by the following factors: Most patients are in the asymptomatic intermediate phase of Chagas disease. Physicians are often not sufficiently trained to recognize this disease in non-endemic countries, and the lack of awareness can lead to missed diagnostic opportunities. Active screening programs for high-risk populations in Japan are therefore required to enable early diagnosis and treatment strategies for Chagas disease. In the past decade, to prevent transmission by blood transfusion, a questionnaire has been used to screen Latin Americans who choose to donate blood in Japan. However, this system does not enable early diagnosis and treatment of patients with Chagas disease [3]. Moreover, there are unfortunately no screening programs for high-risk populations including organ transplant patients, pregnant women, and neonates in Japan.

Based on the relatively high prevalence (> 0.05%) of Chagas disease in European countries, systemic screening for Chagas disease in asymptomatic Latin American residents is considered a cost-effective public health strategy [12]. Therefore, careful consideration based on accurate surveillance is needed to establish an active screening strategy in Japan. Although the prevalence of Chagas disease among at-risk blood donors was previously reported to be 0.017%, this was not necessarily reflective of the prevalence because of sample selection bias [3]. Legal authorities for Chagas disease reporting systems can be helpful for obtaining information about the prevalence and clinical characteristics and contributing to the public policy-making process. In addition, it is desirable that the target population of active screening be covered by national health insurance with regard to the return on investment of public health programs.

Anti-trypanosomal treatments for patients with chronic Chagas disease are controversial, but the early treatment of asymptomatic patients is considered essential to prevent local transmission and Chagas cardiomyopathy. Furthermore, the risk of drug toxicity is higher in older individuals than in younger adults; hence, anti-trypanosomal treatments for patients older than 50 years are not recommended [13]. The Benznidazole Evaluation for Interrupting Trypanosomiasis (BENEFIT) trial, which was the first randomized double-blind controlled clinical trial to investigate the role of benznidazole in patients with chronic Chagas cardiomyopathy, showed that anti-trypanosomal treatment for patients with Chagas cardiomyopathy did not reduce clinical cardiac deterioration [14]. Therefore, this study points to the importance of early diagnosis and treatment of patients with asymptomatic Chagas disease to prevent the progression of cardiomyopathy. Early diagnosis and treatment also contribute to the prevention of vertical transmission. Serum parasite load is significantly reduced in patients treated with anti-trypanosomal drugs [14], and several reports have shown that anti-trypanosomal treatment of infected women of reproductive age can also be an effective strategy to prevent congenital transmission of Chagas disease [15, 16].

Our results do not accurately reflect the full clinical characteristics and prevalence of Chagas disease across Japan because of sample selection bias and the small sample size. However, our study showed that patients with Chagas disease living in Japan may miss opportunities for treatment because of substantial diagnostic delays. A large sample size will be required to obtain a more accurate evaluation of the prevalence of Chagas disease and an economic evaluation of systematic screening programs for the Latin American population.

## Conclusions

This report represents the first step towards an epidemiological study of Chagas disease in Japan. Further large-scale epidemiological studies on the prevalence of Chagas disease may help to determine the need for systematic screening programs for the Latin American population.

## Data Availability

Data are available from the corresponding author on reasonable request.
